# The Story of the Insane from Year to Year

**Published:** 1901-09-28

**Authors:** 


### THE STORY OF THE INSANE FROM YEAR TO YEAR.

(Thirteenth Series.)

### WEST RIDING ASYLUM AT SHEFFIELD.

Not fewer than the names of 1,634 patients were on the books of this asylum on January 1st, 1899, and by December 31st this number had risen to 1,663. There were 414 first admissions, 86 readmissions, 211 discharged recovered, 52 discharged relieved, 17 discharged not improved, and 192 died. The average number resident during the year 1,650, and the total number of persons under treatment was 2,112. Calculated on the admissions the recoveries stand at 42·2 per cent., and calculated on the average number resident the deaths stand at 11·3, both of these percentages being a little above the average. Of the deaths during the year 101 were caused by cerebral and spinal diseases, 18 by consumption, 7 by pneumonia, 4 by bronchitis, 2 by enteritis, and 1 by typhoid fever. In 285 instances moral causes, such as domestic trouble, adverse circumstances and religion are credited with having had something to do with the production of the insanity; intemperance in drink accounts for 80 of the year's admissions, and hereditary influence for 101. The asylum is somewhat overcrowded, and Dr. Kay refers to the large number of helpless cases under treatment, and he thinks, considering these facts, that the general health was fairly good. Enteric fever made its appearance—six patients were affected and one died. Lectures and practical instruction are given by the medical officers to the attendants and nurses, and six of the former and two of the latter obtained the nursing certificate of the Psychological Association. During the year the clerk of the asylum and three of the attendants received pensions. The medical staff consists of medical superintendent and five assistant medical officers—one of the latter being a lady. The weekly rate of maintenance is a fraction over 9s. per head.

### GLASGOW DISTRICT ASYLUM AT WOODILEE.

Unfortunately the statistics are not for the year ending December 31st, 1899, but for the year ending May 15th, 1900. On May 15th, 1899, the asylum contained 734 patients, and on May 15th, 1900, there were 753 in residence. The first admissions were 157, the readmissions 76, the recoveries 90, the deaths 60. The daily average number resident was 737, and the highest number resident on any one day was 755. The percentage of recoveries calculated on the admissions was 38·6, and the percentage of deaths calculated on the average number resident was 8·1. Of the deaths during the year, 28 were due to cerebral and spinal diseases, 10 to consumption, and 4 to pneumonia. Of the year's admissions various moral influences are supposed to have been the cause of the insanity in 11 cases, intemperance in drink in 41, previous attacks in 14, and hereditary influence in 27. Dr. Blair evidently thinks the percentage of cases due to alcoholic intemperance is lower than usual, and he remarks that "a number of the acuter forms of insanity from this cause continue to be treated to a conclusion in the observation wards of the poorhouse;" but it must always be very difficult to ascertain whether drink is the sole cause of insanity in any given case. As in other asylums the old cry is heard of short service among the attendants and nurses, and an attempt to lengthen that service is to be made by giving higher pay and by shortening the periods on duty. We wish Dr. Blair success but even if these concessions should fail in their object, they are nevertheless very laudable. The cost per head per annum was £23 13s. 1d.

### LONDON COUNTY ASYLUM AT COLNEY HATCH.

This asylum contained 2,556 patients on January 1st, 1899, and 2,543 on December 31st. There were 465 first admissions, 126 readmissions, 245 recoveries, 119 discharged relieved, and 240 deaths. The average number resident during the year was 2,543, and the total number of persons under care was 3,147. Excluding transfers and calculating the recoveries on the number of the year's admissions they stand at 46·12 per cent., and calculating the deaths on the average number resident they stand at 9·43 per cent. Of the year's deaths 124 were caused by cerebral and spinal diseases, 23 by consumption, 10 by pneumonia, 3 by bronchitis, 1 by pleurisy, 15 by colitis, and 1 by enteritis. Of the year's admissions the insanity is supposed to have been caused by such moral causes as domestic trouble, mental anxiety, religion, &c., in 152 instances, by intemperance in drink in 105, by hereditary influence in 162, and by "previous attacks" in 175. In the large number of 113 no cause is given. Enteric fever broke out during the year, and there were 9 cases, two ending in death. A large number of the women were attacked by colitis, and, as already pointed out, 15 died (Dr. Seward gives the number at 18, so there is probably a mistake in the compilation of the table on p. 157). "Although the disease used frequently to be prevalent, it is ten years since an epidemic occurred here," and Dr. Seward continues, "no sanitary defect could be detected which could account for the outbreak, and it is remarkable that a large proportion of the cases occurred in the new buildings where the wards are particularly well ventilated, and there is a very liberal allowance of cubic space for each patient." Dr. Seward says that one case in a ward was frequently soon followed by others in the same ward, and he thinks that this fact points towards infection. At first sight it seems like it; but how account for the first case, and may not all the cases in the same ward be due to the same cause, whatever it is, and the time between the notification of the cases be due either to delayed incubation or to delay in the microbes finding their way into the alimentary canal? At any rate isolation of the first cases, although eminently desirable, is not always followed by cessation of the epidemic; but then it may always be said that the separation of the diseased from the healthy did not take place soon enough. It has been often said that this form of dysentery is not much known out of asylums, which is rather curious, and it is more curious still that it was not much known thirty years ago in asylums. The committee record their "appreciation of the continued zeal displayed by Dr. Seward and the staff generally in the discharge of their duties." The weekly cost of maintenance was 9s. 8d. per head.

